# Visualizing host-virus interactions at high resolutions *in situ*

**DOI:** 10.1063/4.0000991

**Published:** 2025-10-27

**Authors:** Yong Xiong

**Affiliations:** Yale University, New Haven, CT, USA

## Abstract

Understanding virus and macromolecular structures in their natural viral and cellular environments is key to advancing biology and improving health. Here, I highlight recent advancements in *in situ* single-particle cryo-electron microscopy (cryo-EM), which has enabled us to observe how HIV-1 capsids interact with antiviral compounds directly within the virus’s native environment. Additionally, we showcase the unprecedented detail of protein synthesis in human cells using a method that combines automated cryo-focused ion beam (FIB) milling with *in situ* single-particle cryo-EM. With this approach, we resolved a 2.19 Å resolution structure of the human 80S ribosome and uncovered 23 distinct functional states. We also obtained high-resolution structural insights from cells treated with the anti-tumor drug homoharringtonine and the inhibitor cycloheximide. Collectively, our work represents a significant advancement in structural studies within natural contexts, highlighting a powerful tool to investigate virus infection and basic biology.

